# Hydrogen Sulfide and/or Ammonia Reduces Spermatozoa Motility through AMPK/AKT Related Pathways

**DOI:** 10.1038/srep37884

**Published:** 2016-11-24

**Authors:** Yong Zhao, Wei-Dong Zhang, Xin-Qi Liu, Peng-Fei Zhang, Ya-Nan Hao, Lan Li, Liang Chen, Wei Shen, Xiang-Fang Tang, Ling-Jiang Min, Qing-Shi Meng, Shu-Kun Wang, Bao Yi, Hong-Fu Zhang

**Affiliations:** 1 State Key Laboratory of Animal Nutrition, Institute of Animal Sciences, Chinese Academy of Agricultural Sciences, Beijing 100193, China; 2 College of Chemistry and Pharmaceutical Sciences, Qingdao Agricultural University, Qingdao 266109, P. R. China; 3 Key Laboratory of Animal Reproduction and Germplasm Enhancement in Universities of Shandong, Qingdao 266109, P. R. China

## Abstract

A number of emerging studies suggest that air pollutants such as hydrogen sulfide (H_2_S) and ammonia (NH_3_) may cause a decline in spermatozoa motility. The impact and underlying mechanisms are currently unknown. Boar spermatozoa (*in vitro*) and peripubertal male mice (*in vivo*) were exposed to H_2_S and/or NH_3_ to evaluate the impact on spermatozoa motility. Na_2_S and/or NH_4_Cl reduced the motility of boar spermatozoa *in vitro.* Na_2_S and/or NH_4_Cl disrupted multiple signaling pathways including decreasing Na^+^/K^+^ ATPase activity and protein kinase B (AKT) levels, activating Adenosine 5′-monophosphate (AMP)-activated protein kinase (AMPK) and phosphatase and tensin homolog deleted on chromosome ten (PTEN), and increasing reactive oxygen species (ROS) to diminish boar spermatozoa motility. The increase in ROS might have activated PTEN, which in turn diminished AKT activation. The ATP deficiency (indicated by reduction in Na^+^/K^+^ ATPase activity), transforming growth factor (TGF_β_) activated kinase-1 (TAK1) activation, and AKT deactivation stimulated AMPK, which caused a decline in boar spermatozoa motility. Simultaneously, the deactivation of AKT might play some role in the reduction of boar spermatozoa motility. Furthermore, Na_2_S and/or NH_4_Cl declined the motility of mouse spermatozoa without affecting mouse body weight gain *in vivo.* Findings of the present study suggest that H_2_S and/or NH_3_ are adversely associated with spermatozoa motility.

A number of recent publications have reported a decline in spermatozoa motility^1^,^2^,^3^,^4^,^5^. Other parameters of human spermatozoa quality have also decreased during the past few decades^3^,^6^. Along with diminishing spermatozoa quality, the incidence of infertility has increased from 7–8% in 1960 to a current level of 20–35%^3^,^7^; however, the decline in spermatozoa quality is not fully understood. Environmental pollutants including gases and particulate matter (PM) from a variety of sources^8^, which emanate from vehicles, industry, power stations, livestock production systems, and natural processes^9^,^10^ have been considered as a reason for decreasing spermatozoa quality^3^,^5^,^6^,^11^,^12^. These pollutants damage ecological systems and adversely affect public health^12^,^13^,^14^. PM has been of concern because epidemiological findings link them to a growing list of adverse health effects^8^,^15^. Ozone, carbon monoxide (CO), sulfur dioxide (SO_2_), and nitrogen oxides (NO_x_) are considered as the main gaseous materials in air pollution^4^,^14^. However, there are more than 50 kinds of volatile organic compounds such as ammonia (NH_3_) and hydrogen sulfide (H_2_S) in air pollution that are free or bound to PM^16^,^17^. PM can carry large amounts of NH_3_ molecules for long time periods (up to 7 μg NH_3_ per mg of respirable PM), and significantly higher amounts of H_2_S have been detected in PM from 5 to 20 μm in diameter than in larger particles in the range of 20–75 μm^9^,^16^,^17^.

NH_3_, the most abundant alkaline gas in the atmosphere, is predominantly a product of animal husbandry and NH_3_-based fertilizer application; it is also a major component of total reactive nitrogen^18^. In physiological conditions, NH_3_ is mostly present as ammonium (NH_4_^+^) in serum, which is converted to urea in the liver and then excreted by the kidneys to maintain low serum levels (<50 μM in adults)^19^. Global NH_3_ emissions have been increasing over the last few decades, which raises concerns that it might negatively influence environmental and public health as well as climate change due to the role of NH_3_ in PM formation, visibility degradation, and atmospheric deposition of nitrogen within ecosystems^18^,^20^,^21^. H_2_S, a colorless, flammable, and foul odored gas, has been considered poisonous for a long time^10^,^22^. Recently, it has been found to be a signaling molecule regulating insulin secretion/sensitivity and vascular homeostasis to control blood pressure and penile tone^10^,^22^,^23^. Furthermore, it is a mediator of dietary restriction^24^. The concentration of H_2_S in blood has been detected to be ~50 μM in animals and ~600 μM in asthmatic patients^25^. Although H_2_S acts as a transmitter in biological systems, its poisonous nature is still a problem because it is the predominant sulfur contaminant of natural gas with widespread environmental and occupational exposure from industrial activities^22^,^26^.

Although NH_3_ and H_2_S have not been considered as major gaseous pollutants, they are present in air, carried by PM, and strikingly little is understood about their effects on spermatozoa quality and the underlying mechanisms. Therefore, this current investigation aimed to explore the impact of NH_3_ and H_2_S on spermatozoa quality and the underlying mechanisms.

## Results

### Na_2_S and a combination of NH_4_Cl + Na_2_S decreased boar spermatozoa motility

**Figure 1:**
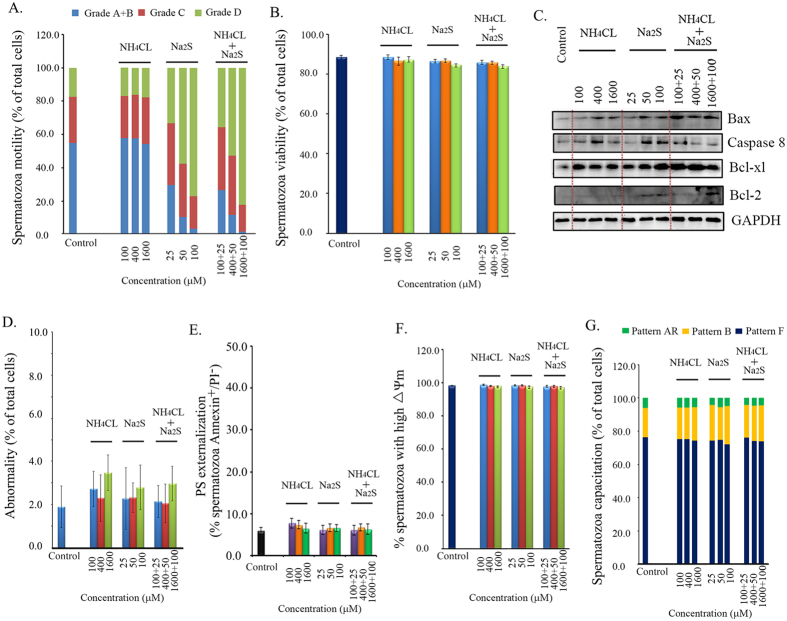
(**A**) Spermatozoa motility determined by CASA. Y-axis = % of total cells, X axis = the treatment concentration (μM). n = 6–8. **(B)** Spermatozoa viability. Y-axis = % of total cells, X-axis = the treatment concentration (μM). n = 6–8. **(C)** Protein levels of apoptotic markers detected by Western blotting. n = 3. **(D)** The abnormality of boar spermatozoa detected by eosin Y staining. n = 5. **(E)** Spermatozoon plasma membrane phosphatidylserine (PS) externalization. n = 4. **(F)** The mitochondrial membrane potential (△Ψm) was measured by the specific probe JC-1 using flow cytometry. Y-axis = % of total cells with high membrane potential, X-axis = the treatment concentration (μM) n = 4. **(G)** Spermatozoa capacitation status detected by CTC staining. Y-axis = % of total cells, X axis = the treatment concentration (μM). n = 4.

To examine the effects of NH_4_Cl and/or Na_2_S on boar spermatozoa motility, we treated the spermatozoa with different concentrations of NH_4_Cl, Na_2_S, and a combination of NH_4_Cl + Na_2_S in spermatozoa incubation medium for 24 h. Treatments involving 100, 400, and 1600 μM NH_4_Cl did not alter spermatozoa motility. However, 25, 50, and 100 μM Na_2_S treatments and a combination of NH_4_Cl + Na_2_S significantly decreased spermatozoa motility as shown by a dramatic increase in grade D spermatozoa (immotile) and a decrease in grade A + B spermatozoa in a concentration dependent manner [Fig. 1A; *p* < 0.05 (grade A + B, grade D): Na_2_S treatment compared to control treatment, Na_2_S + NH_4_Cl treatment compared to control treatment]. Immotile spermatozoa (grade D) increased from 17% in the control to 77% in the Na_2_S-100 μM treatment and 82% in the NH_4_Cl-1600 μM + Na_2_S-100 μM treatment. The straight motile spermatozoa (grade A + B) decreased from 55% in the control to 3% in the Na_2_S-100 μM treatment and 1% in the NH_4_Cl-1600 μM + Na_2_S-100 μM treatment.

To assess boar spermatozoa viability after 24 h treatments, we analyzed the living cells with a Live/Dead Sperm Viability Kit using flow cytometry. As presented in Fig. 1B, none of the treatments changed spermatozoa viability. Most treatments increased the protein levels of apoptosis markers Bax and Caspase 8; at the same time, the treatments also elevated the anti-apoptosis markers Bcl-xl and Bcl-2 (Fig. 1C). Boar spermatozoa abnormality rate was very low (2–4% of total cells) and no treatments affected these levels (Fig. 1D). Phosphatidylserine (PS) externalization at the spermatozoon plasma membrane is a process that indicates plasma membrane scrambling. We further investigated the effect of NH_4_Cl and/or Na_2_S on PS externalization at the spermatozoon plasma membrane. The level of PS externalized in spermatozoa plasma membranes was very low (<10% of total viable spermatozoa) and no treatments disrupted PS externalization (Fig. 1E). To determine the function of mitochondria, we evaluated the spermatozoon mitochondrial membrane potential (△Ψm). No treatments affected the population of spermatozoa presenting high △Ψm (Fig. 1F), which remained at around 97%. Subsequently, the effects of NH_4_Cl and/or Na_2_S on spermatozoa capacitation were assessed by CTC staining. As reported in Fig. 1G, NH_4_Cl and/or Na_2_S had no evident effects on the percentage of cells showing the F pattern (typical of freshly ejaculated cells), B pattern (typical of capacitated cells), or AR pattern (typical of acrosome-reacted cells).

### Na_2_S and the combination of NH_4_Cl + Na_2_S increased ROS formation in boar spermatozoa

**Figure 2:**
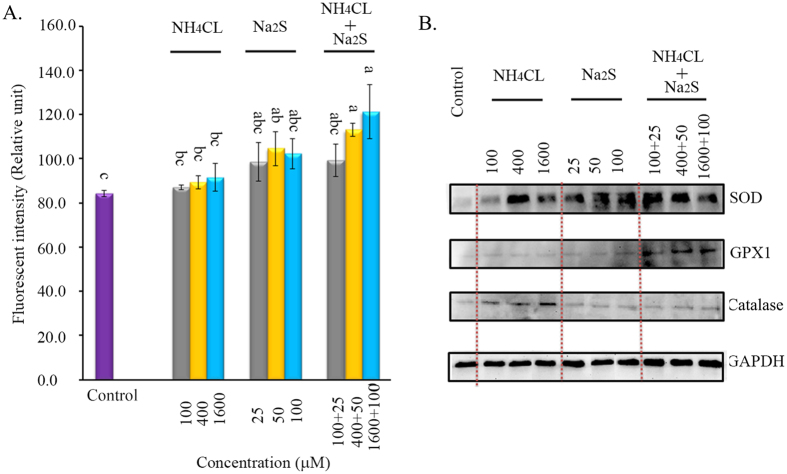
(**A**) ROS levels in all the treatments by H_2_DCFDA kit using flow cytometry. Y-axis = the fluorescent intensity (relative unit). X-axis = the treatment concentration (μM). ^a,b,c^ Means not sharing a common superscript are different (*p* < 0.05). n = 3. **(B)** Protein levels of SOD, GPX, and catalase detected by Western blotting. n = 3.

To investigate the effects of NH_4_Cl and/or Na_2_S on ROS production, we analyzed the ROS levels (H_2_O_2_) in spermatozoa using flow cytometry. Na_2_S and the combination of NH_4_Cl + Na_2_S increased the level of H_2_O_2_ after 24 h of treatment. Na_2_S-50 μM, NH_4_Cl-400 μM + Na_2_S-50 μM, and NH_4_Cl-1600 μM + Na_2_S-100 μM significantly increased H_2_O_2_ levels by 20–40% (Fig. 2A, *p* < 0.05). At the same time, the anti-oxidant enzymes catalase, total-SOD (super oxide dismutase), and GPX1 (glutathione peroxidase) were determined (Fig. 2B). All treatments increased SOD protein levels in spermatozoa. The combination of NH_4_Cl and Na_2_S elevated the protein levels of GPX1; however, NH_4_Cl or Na_2_S alone did not affect GPX1. NH_4_Cl treatments elevated the protein level of catalase; however, Na_2_S or combinations of NH_4_Cl and Na_2_S treatments did not affect catalase protein levels.

### Na_2_S and/or NH_4_Cl decreased ATPase, increased TAK1, and activated AMPK in boar spermatozoa

**Figure 3:**
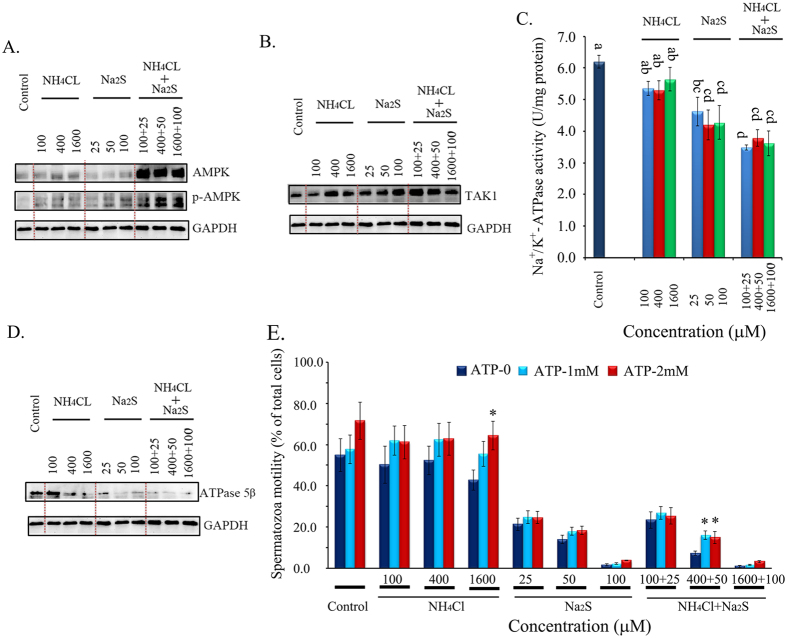
Protein levels of AMPK and p-AMPK (n = 3) **(A),** TAK1 (n = 3) **(B)** detected by Western blotting. **(C)** Total ATPase activity measured by spectrophotometry. Y-axis = the activity (U/mg protein), and X-axis = the treatment concentration (μM). ^a,b,c^ Means not sharing a common superscript are different (*p* < 0.05). n = 4. **(D)** Protein levels of ATPase 5β detected by Western blotting. n = 3. **(E)** ATP addition on spermatozoa motility. Y-axis = % of total cells, X-axis = the treatment concentration (μM). *Means significant at *p* < 0.05 compared to the same treatment without ATP addition (ATP-0). n = 3.

The AMP-activated protein kinase (AMPK) pathway plays a crucial role in boar spermatozoa motility. The combination of NH_4_Cl and Na_2_S treatments dramatically increased AMPK protein levels by ~5-fold (Fig. 3A). The phosphorylated form (Thr^172^) of AMPK was elevated by NH_4_Cl alone, Na_2_S alone, and the combination of NH_4_Cl + Na_2_S treatments (Fig. 3A). Moreover, the protein levels of the phosphorylated form (Thr^172^) of AMPK in the NH_4_Cl + Na_2_S combination treatments were higher than those in the treatments of NH_4_Cl or Na_2_S alone. The NH_4_Cl + Na_2_S treatments synergistically increased the phosphorylated form (Thr^172^) of AMPK (Fig. 3A). AMPK can be activated through the TAK1, CaMKK, or LKB1 pathways, or by changing the ratio of AMP/ATP. NH_4_Cl, Na_2_S, and combinations of NH_4_Cl + Na_2_S increased the protein levels of TAK1 (Fig. 3B); however, none of the treatments altered CaMKKα/β, and Na_2_S, furthermore, the combination of NH_4_Cl + Na_2_S treatment decreased LKB1 level (data not shown). The activity of Na^+^/K^+^-ATP synthesis enzymes (Na^+^/K^+^-ATPase) was decreased by Na_2_S and the combination NH_4_CL + Na_2_S treatments. The activity of Na^+^/K^+^-ATPase in the combination treatments was even lower than that in Na_2_S treatments (Fig. 3C). Although NH_4_Cl treatments decreased Na^+^/K^+^-ATPase activity compared to the control treatment, the difference was not significant. The data were confirmed by the protein level of ATPase5β, which was decreased by Na_2_S and the combination NH_4_Cl + Na_2_S treatments in Western blotting analysis (Fig. 3D).

### ATP addition partially rescued boar spermatozoa motility

To test whether ATP deficiency was the major reason for the decrease in boar spermatozoa motility caused by Na_2_S and/or NH_4_Cl, ATP rescue experiments were performed with addition of ATP. The addition of ATP only slightly elevated spermatozoa motility. NH_4_Cl-1600 + ATP-2 mM elevated the percentage of grade A + B motility compared to the NH_4_Cl-1600 treatment alone (*p* < 0.05). NH_4_Cl-400 + Na_2_S-50 μM + ATP-1 mM and NH_4_Cl-400 + Na_2_S-50 μM + ATP-2 mM increased the percentage of grade A + B motility compared to the NH_4_Cl-400 + Na_2_S-50 μM treatment alone (*p* < 0.05; Fig. 3E).

### Na_2_S and/or NH_4_Cl activated the PI3K, ERK, and PTEN pathways and inhibited the AKT pathway in boar spermatozoa

**Figure 4:**
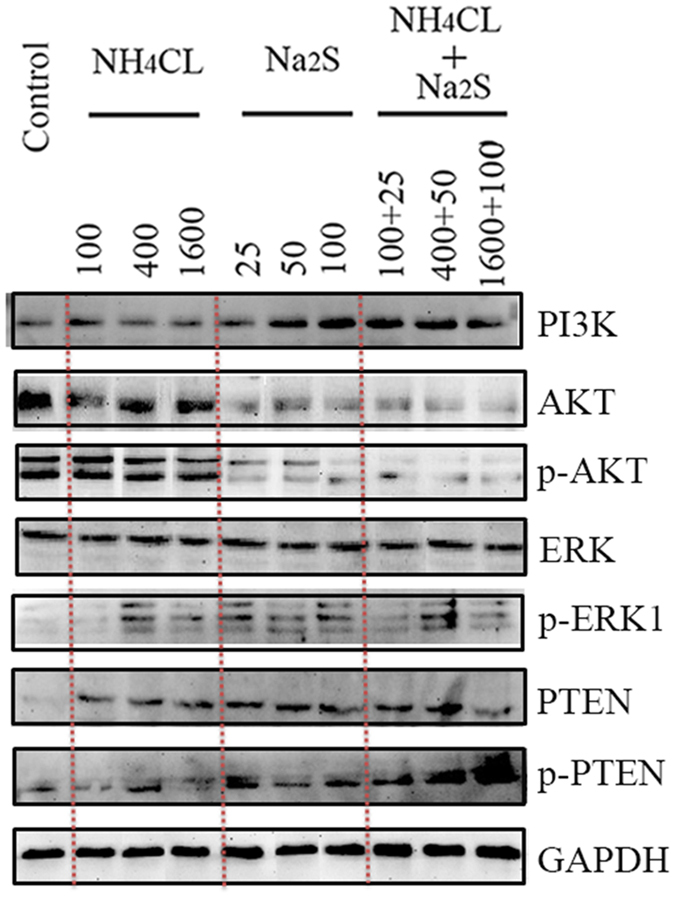
Protein levels of PI3K, AKT, p-AKT, ERK, p-ERK, PTEN, and p-PTEN detected by Western blotting. n = 3.

**Figure 5:**
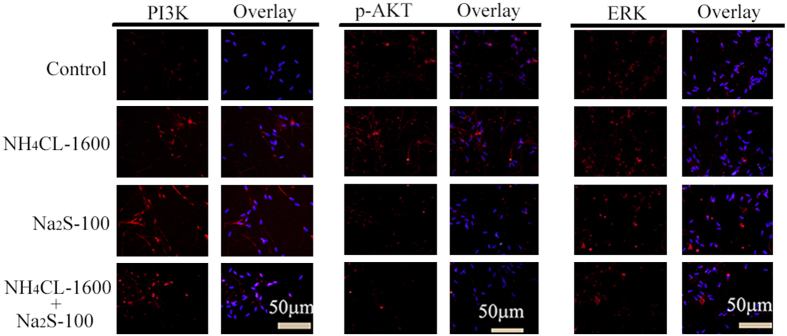
Protein levels of PI3K **(A)**, p-AKT **(B)** and ERK **(C)** detected by IHF. Scale bar = 50 μm. n = 3.

PI3K/AKT/ERK pathways play vital roles in cell biology and the activation of AKT inhibits AMPK activation. The levels of these proteins were determined by Western blotting and immunofluorescent staining. Na_2_S-50 (100 μM) and the combination of NH_4_Cl + Na_2_S treatments increased PI3K protein levels (Figs 4 and 5A). However, Na_2_S and the combination of NH_4_Cl + Na_2_S treatments decreased protein levels of both AKT and the phosphorylated form (Thr^308^) of AKT (p-AKT; Figs 4 and 5B). ERK_1+2_ protein level remained unchanged by all treatments (Figs 4 and 5C). However, NH_4_Cl-400, 1600 μM, Na_2_S-25, 50, 100 μM, and combinations of NH_4_Cl + Na_2_S treatments increased the phosphorylated form (Thr^197^ + Thr^202^) of ERK_1_ (Fig. 4). Phosphatase and tensin homolog deleted on chromosome 10 (PTEN) is an inhibitor for activation of AKT from PI3K. All treatments increased PTEN and the phosphorylated form (Ser^380^ + Thr^382^ + Thr^383^) of PTEN (p-PTEN; Fig. 4).

### Combination of Na_2_S and NH_4_Cl decreased mouse spermatozoa motility *in vivo*

**Figure 6:**
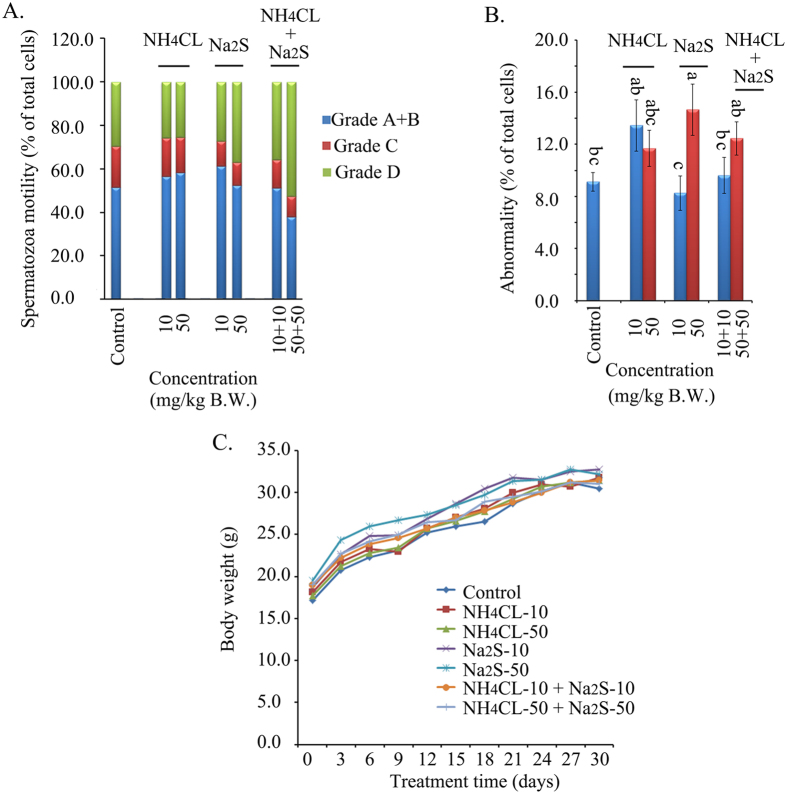
(A) Mouse spermatozoa motility determined by CASA. Y-axis = % of total cells, X-axis = the treatment concentration mg/kg B.W. (*p* < 0.05). n = 8. **(B)** The abnormality of mouse spermatozoa detected by eosin Y staining. ^a,b,c^ Means not sharing a common superscript are different (*p* < 0.05). **(C)** Mouse body weight. Y-axis = body weight (g), X-axis = the treatment time (day). n ≥ 4.

In order to confirm the data obtained from boar spermatozoa *in vitro*, mice were treated with different concentrations of NH_4_Cl and/or Na_2_S (*in vivo* studies). The combination NH_4_Cl-50 mg/kg + Na_2_S-50 mg/kg treatment significantly decreased mouse spermatozoa motility by decreasing the population of grade A + B spermatozoa (Fig. 6A, *p* < 0.05). Furthermore, Na_2_S-50 mg/kg and NH_4_Cl-10 + Na_2_S-10 treatments significantly increased the population of grade D (immotile) spermatozoa (Fig. 6A, *p* < 0.05). Moreover, the Na_2_S-50 mg/kg treatment promoted the number of abnormal mouse spermatozoa (Fig. 6B, *p* < 0.05). Na_2_S-50 + NH_4_Cl-50 mg/kg also elevated spermatozoa abnormalities; however, the difference was not significant at *p* < 0.05. Finally, no treatments disrupted mouse body weight gain during the experimental period (Fig. 6C).

## Discussion

Numerous emerging studies suggest that air pollutants may cause a decline in the motility of human spermatozoa^1^,^2^,^3^,^4^,^5^. NH_3_ and H_2_S are air pollutants that can be free or bound to air PM^8^,^9^,^16^,^17^. In the current study, H_2_S donor Na_2_S and/or NH_3_ donor NH_4_Cl decreased boar spermatozoa motility *in vitro* and reduced mouse spermatozoa motility *in vivo,* which confirmed our hypothesis that air pollutants might reduce spermatozoa motility. However, Na_2_S and/or NH_4_Cl did not change boar spermatozoa viability, abnormality rate, plasma membrane integrity, mitochondrial membrane potential, or capacitation status. This suggests that the toxicity of NH_4_CL and/or Na_2_S treatments (used in current investigation) might be not very high just that spermatozoa motility was declined while the spermatozoa survival was not impaired. This phenomenon was also found by the epidemiology studies^1^,^2^,^3^,^4^,^5^.

Although epidemiological studies have observed that air pollutants decrease spermatozoa motility, the underlying mechanisms are currently unknown. In this investigation, multiple signaling pathways were involved in the reduction of boar spermatozoa motility through the impact of Na_2_S and/or NH_4_Cl. These pathways are all connected to the AMPK pathway. AMPK, an energy status sensor, regulates cellular energy homeostasis, mitochondrial biogenesis and disposal, autophagy, cell polarity, and cell growth and proliferation. AMPK is expressed in the ovaries, testes, and spermatozoa; it regulates gonadal steroidogenesis and is thus involved in fertility^27^. Most interestingly, it modulates spermatozoa motility and other functions^28^,^29^. The increase in AMP: ATP or ADP: ATP ratio can activate AMPK by phosphorylation at Thr^172^ (p-AMPK). It has also been found that Ca^2+^/calmodulin-dependent protein kinase (CaMKK_β_), transforming growth factor (TGF_β_) activated kinase-1 (TAK1), and LKB1 could activate AMPK^28^,^30^. TAK1 can be activated by cytokines. In the current study NH_4_Cl and/or Na_2_S increased TAK1. In line with previous studies where exposure to low levels of airborne irritants stimulated the markers of airway inflammation, where high level of H_2_S were detected in asthma patients, and exposure to different size fractions of PM activated inflammation and oxidative stress signals, Na_2_S and/or NH_4_Cl might elevate inflammatory factors to activate TAK1, which in turn enhances AMPK activation^25^,^31^,^32^. Combinations of Na_2_S + NH_4_Cl significantly elevated the protein levels of AMPK and p-AMPK (Thr^172^) in boar spermatozoa. Na_2_S alone or NH_4_Cl alone stimulated p-AMPK (Thr^172^), but not AMPK protein in boar spermatozoa. Na_2_S and combinations of NH_4_Cl + Na_2_S diminished the activity of Na^+^/K^+^-ATPase in boar spermatozoa. Consistent with the notion that AMPK is very sensitive to the AMP: ATP ratio, our data suggested that NH_4_Cl and/or Na_2_S might decrease ATP production in spermatozoa by inhibiting ATPase, which consequently activated AMPK to reduce spermatozoa motility^28^.

Because boar sperm motility is sensitive to oxidative stress and H_2_O_2_ is the major free radical mediating direct ROS effects in boar spermatozoa, H_2_O_2_ was measured in boar spermatozoa after Na_2_S and/or NH_4_Cl treatments^33^. Interestingly, Nguyen *et al*.^34^ observed that AMPK stimulated intracellular anti-oxidative defense enzymes in chicken spermatozoa. In the current study, Na_2_S and the combination of Na_2_S + NH_4_Cl elevated H_2_O_2_ in boar spermatozoa *in vitro*. However, at the same time the antioxidant enzymes SOD and GPX1 were also increased by N_2_S and the combinations of Na_2_S + NH_4_Cl. These results indicated that N_2_S and the combinations of Na_2_S + NH_4_Cl might promote oxidative stress; on the other hand, in order to defend against the stress, the boar spermatozoa might activate antioxidant systems to suppress ROS. The PI3K/AKT pathways play critical roles in controlling cell survival and spermatozoa motility. Sagare-Patil *et al*.^35^ found that the PI3K-AKT pathway is required for motility and hyper-activation in human spermatozoa and Gallardo Bolaños *et al*.^36^ observed that p-AKT preserved stallion spermatozoa motility. Moreover, activation of AKT inhibits AMPK phosphorylation (Thr^172^)^30^; the PTEN signaling pathway regulates cell proliferation, cell-cycle progression, and cell survival; and ROS, ATP deficiency, and AMPK activation promote PTEN expression and nuclear accumulation^37^,^38^,^39^,^40^. However, PTEN is a negative regulator of AKT. In the current study, Na_2_S and the combinations of Na_2_S + NH_4_Cl increased the protein level of PI3K; however, these treatments decreased AKT and p-AKT levels. Na_2_S and/or NH_4_Cl treatments also elevated the protein levels of PTEN and p-PTEN in boar spermatozoa. This data suggested that the increase in H_2_O_2_, ATP deficiency, and AMPK activation stimulated the PTEN pathway, which consequently inhibited AKT activation. On the other hand, the decrease in AKT stimulates AMPK activation^30^. Furthermore, AKT inhibition might reduce spermatozoa motility due to its importance in controlling spermatozoa motility^35^,^36^. PI3K/ERK pathway is very important for cell survival and growth too^35^,^41^. PI3K might activate ERK pathway to increase p-ERK in the spermatozoa. It also was found that spermatozoa viability was not altered by NH_4_CL and/or Na_2_S treatment. Therefore p-ERK might play crucial role in the sperm survival.

It is known that mammalian spermatozoa are transcriptionally and translationally inactive. However, it has been found that post-translational modifications and protein acquisition/degradation play very import role in spermatozoa in order to response to the changes in the epididymis and female tract^42^. In current study, NH_4_CL and/or Na_2_S treatments altered many proteins or phosphorylated proteins in spermatozoa which might be due to the post-translational modifications and protein acquisition/degradation.

The combination of Na_2_S-50 mg/kg + NH_4_Cl-50 mg/kg treatment significantly decreased mouse spermatozoa motility by reducing the percentage of grade A + B spermatozoa and elevating grade D (immotile) spermatozoa *in vivo,* which agreed with the data from boar spermatozoa *in vitro*. The data further indicated that exposure to one component of air pollution may not be excessively problematic; however, a combination of two or more components of air pollution might synergistically pose problems to human health.

**Figure 7:**
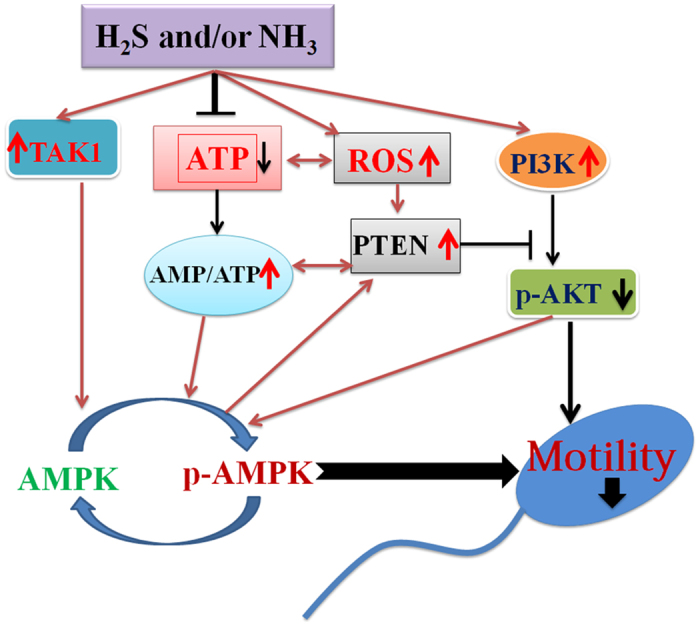
H_2_S and/or NH_3_ regulation of spermatozoa motility through multiple signaling pathways.

In conclusion, the data from boar spermatozoa *in vitro* demonstrated that H_2_S and/or NH_3_ disrupted multiple signaling pathways to diminish spermatozoa motility. The main points include a decrease in ATP production and AKT levels, activation of AMPK and PTEN, and an increase in ROS. The increase in ROS might activate PTEN, which in turn diminished AKT activation. The ATP deficiency (indicated by reduction in Na^+^/K^+^ ATPase activity), TAK1 activation, and AKT deactivation stimulate AMPK, which results in a decline in spermatozoa motility (Fig. 7). And the *in vivo* data with mouse spermatozoa confirmed the *in vitro* results. Findings of the present study suggest that H_2_S and/or NH_3_ may be adversely associated with spermatozoa quality, particularly spermatozoa motility.

## Materials and Methods

### Collection of boar semen and preparation of spermatozoa samples for different treatments

Porcine spermatozoa incubation medium powder was purchased from Hangzhouyuefeng Bio-engineering CO., Ltd. (Hangzhou, China). The medium can maintain pH, osmolarity, ion balance, and buffering to sustain spermatozoa activity for 7–14 d. Semen samples from Duroc boars (2–3 y old) were commercially obtained from a Regional Porcine Company (Hengshengyuan CO., Ltd., Qingdao, China). All boars were housed in individual pens in an environmentally controlled building (15–25 °C) according to Regional Government and national regulations. Artificial insemination took place using preserved liquid semen from boars of demonstrated fertility. Fresh ejaculates were collected with the gloved hand technique and stored at 17 °C before use^28^. Semen samples from 3–5 animals were pooled each time to minimize individual boar variation and the samples had >80% morphologically normal spermatozoa. Subsequently, the semen was diluted with incubation medium to a concentration ~40 × 10^6^ cell/ml and then the cells were treated with NH_3_ donor NH_4_Cl (Cat #:09718; Sigma-Aldrich Co. LLC. St. Louis, MO, USA) and/or H_2_S donor Na_2_S (Cat #:431648; Sigma-Aldrich Co. LLC.)^43^,^44^. There were ten treatment groups: (1) Vehicle control (blank incubation medium); (2) NH_4_Cl-100 μM; (3) NH_4_Cl-400 μM; (4) NH_4_Cl-1600 μM; (5) Na_2_S-25 μM; (6) Na_2_S-50 μM; (7) Na_2_S-100 μM; (8) H_4_Cl-100 μM + Na_2_S-25 μM; (9) NH_4_Cl-400 μM + Na_2_S-50 μM; (10) NH_4_Cl-1600 μM + Na_2_S-100 μM. The treatment time was 24 h.

### Mouse exposure to NH_4_CL and/or Na_2_S

This investigation was carried out in strict accordance with the recommendations in the Guide for the Care and Use of Laboratory Animals of the National Institutes of Health. The protocol was approved by the Committee on the Ethics of Animal Experiments of Qingdao Agricultural University IACUC (Institutional Animal Care and Use Committee). Animals were housed under a light: dark cycle of 12:12 h and at a temperature of 23 °C and humidity of 50–70%. Mice were handled humanely during the experiments and in order to minimize fighting, two mice were housed per shoebox-type cage with a solid floor and woodchip bedding. Mice had constant access to food (chow diet) and water, and bedding was changed every other day^45^.

Mice were exposed to NH_4_Cl and/or Na_2_S via oral gavage. The NH_4_Cl and/or Na_2_S dosing solution was freshly prepared on a daily basis in phosphate buffered saline (PBS) solution and given to animals as described previously^24^,^46^. There were 7 treatments (8 mice/treatment): (1) vehicle control (PBS); (2) NH_4_Cl-10 mg/kg BW; (3) NH_4_Cl-50 mg/kg BW; (4) Na_2_S-10 mg/kg BW; (5) Na_2_S-50 mg/kg BW; (6) NH_4_Cl-10 mg/kg + Na_2_S-10 mg/kg BW; (7) NH_4_Cl-50 mg/kg BW + Na_2_S-50 mg/kg BW. The volume of gavage for each mouse was 0.1 ml/d. The gavage took place every morning for 30 d starting at 25 d of age.

### pH measurement

The pH of the samples was determined by a pH-meter (PB-10; Satorium; Germany). The electrode was thoroughly washed every time with distilled water before and after sample detection. 3–5 replicates were assessed for every sample^47^.

### Evaluation of spermatozoa motility by computer-assisted sperm analysis system

Spermatozoa motility was assessed by the computer-assisted sperm assay (CASA) method according to World Health Organization guidelines^48^. After 24 h of treatment, boar spermatozoa were incubated at 37.5 °C for 30 min then samples were placed in a pre-warmed counting chamber (MICROPTIC S.L., Barcelona, Spain). After euthanized, spermatozoa was collected from cauda epididymis of mice and suspended in DMEM/F12 medium with 10% FBS and incubated at 37.5 °C for 30 min then samples were placed in a pre-warmed counting chamber^49^. The Micropic Sperm class analyzer (CASA system) was used in this investigation. It was equipped with a 20-fold objective, a camera adaptor (Eclipse E200, Nicon, Japan), and a camera (acA780-75gc, Basler, Germany), and it was operated by an SCA sperm class analyzer (MICROPTIC S.L.). The classification of sperm motility was as follows: grade A linear velocity >22 μm s^−1^; grade B <22 μm s^−1^ and curvilinear velocity >5 μm s^−1^; grade C curvilinear velocity <5 μm s^−1^; and grade D immotile spermatozoa^48^.

### Morphological observations of spermatozoa

After 24 h of treatment and subsequent incubation at 37.5 °C for 30 min, the boar spermatozoa were stained with Eosin Y (1%) as described by Shin *et al*.^50^. Briefly, the extracted caudal epididymis from mice were placed in RPMI and finely chopped and then Eosin Y (1%) was added for staining. Spermatozoa abnormalities were classified into head or tail morphological abnormalities: two heads, two tails, blunt hooks, and short tails (for each treatment group sample, 3–6 repeats).

### Flow cytometry analysis

A FACSCalibur^TM^ flow cytometer (BD Bioscience, Mississauga, ON) containing a 488-nm laser, forward scatter (FSC) diode detector, and photomultiplier tube (PMT) SSC detector was used. Data collection was performed with CellQuest software and further analyzed with ModiFit LT software (ModiFit LT for Maclntel). About 10 000–20 000 spermatozoa were analyzed in each group^28^.

### Analysis of boar spermatozoa viability by flow cytometry

As described by Hurtado de Llera *et al*.^28^, fluorescent staining using the Live/Dead spermatozoa viability kit (Cat #: L7011; Thermo Fisher scientific Inc., Waltham, MA, USA) was performed to measure boar spermatozoa viability following the manufacturer’s instructions. Briefly, 5 μl of SYBR-14 (20 μmol/l) was added to 1 ml of spermatozoa sample in PBS with 40 × 10^6^ cells/ml and incubated for 10 min at room temperature (RT) in darkness. And then 10 μl of propidium iodide (PI; 2.4 mM) was added into the sample and incubated for another 10 min at RT in darkness. After incubation, spermatozoa were analyzed by the flow cytometer. The fluorescence values of SYBR-14 were collected in the FL1 sensor using a 525 nm bad pass filter, whereas PI fluorescence was collected in the FL3 sensor using a 620 nm bad pass filter. The viable spermatozoa were expressed as the average of the percentage of SYBR14^+^/PI^−^ spermatozoa ± SEM.

### Evaluation of phosphatidylserine externalization at the outer leaflet plasma membrane of boar spermatozoa by flow cytometry

The spermatozoa plasma membrane phosphatidylserine (PS) externalization was detected by Annexin-V-FITC (Cat #: FA101; Beijing TransGen Biotech Co., Ltd.; Beijing, China) to specifically detect PS translocation from the inner to the outer leaflet of the boar spermatozoa plasma membrane as described by Hurtado de Llera *et al*.^28^. Briefly, after 24 hr treatment, boar spermatozoa cells were collected and resuspended in 1× Annexin V binding buffer (100 μl). Then 5 μl Annexin V and 5 μl PI were added into the samples following by incubation for 15 min in the darkness at RT. After incubation, 400 μl of binding buffer were added to each sample and mixed before flow cytometry analysis. The fluorescence values of probes Annexin V-FITC and PI were collected in the FL1 and FL3 sensors using a 520 and 620 nm bad pass filter, respectively. The results are expressed as the average of the percentage of Annexin V^+^/PI^−^ spermatozoa ± SEM.

### Analysis of boar spermatozoa mitochondrial membrane potential (△Ψm) by flow cytometry

The mitochondrial membrane potential (△Ψm) was measured by the specific probe JC-1 (5,5′,6,6′-tetrachloro-1,1′,3,3′ tetraethylbenzymidazolyl carbocyanine iodine; JC assay kit, Cat #: M34152; Thermo Fisher scientific Inc., Waltham, MA, USA)^28^. Briefly, after 24 hr treatment, the sperm cells was collected and resuspended in 1 ml PBS, and 10 μl of 200 μM JC-1 solution was added into the samples following incubation at 37 °C for 30 min. The samples were analyzed by flow cytometry analysis. The fluorescence value was collected using a 525 nm bad pass filter and the percentage of orange stained cells was recorded to be the population of spermatozoa with a high mitochondrial membrane potential. The data were present as the average percentage of high △Ψm spermatozoa ± SEM.

### Determination of capacitation status

Sperm cells were tested with chlortetracycline (CTC; Cat #: 16663-5; Cayman Chemical, Ann Arbor, Michigan, USA) assay to assess the capacitation status as well as acrosome reaction as described by Bucci *et al*.^51^. Briefly, 100 μL of sperm suspension (40 × 10^6^spermatozoa/ml) was mixed with 100 μL of CTC solution (750 μM CTC; 5mM L-cysteine; 130 mM NaCl; 20 mM TrisHCl) and 200 μL of 12.5% glutaraldehide solution and incubation for 30 sec at RT in darkness. 20 μL of the solution was spotted onto a slide and mounted with Vecta shield mounting medium (Vector Laboratories, Burlingame, CA, USA). The sperm cells were viewed by a fluorescence microscope. Three different patterns were identified:

F:fluorescence in the whole head, typical of freshly ejaculated spermatozoa;

B:fluorescence in the acrosomal region and negativity of the post-acrosomal region, typical of capacitated spermatozoa;

AR:fluorescence in the equatorial line, typical of acrosome reacted cells.

At least 500 cells were counted in each treatment group.

### Detection of boar spermatozoa intracellular levels of H_2_O_2_

20, 70-dichlorodihydrofluoresceindiacetate (H_2_DCFDA; Cat #: E004; Nanjing Jiancheng Biotech Inc., Nanjing, China) was used to detect H_2_O_2_ as described by Awda *et al*.^33^. After 24 hr treatment, the sperm cells were collected and staining with H2DCFDA (10 μM) in darkness for 30 min, then fluorescence was assessed in a FACS Calibur flow cytometer (BD Bioscience, Mississauga, ON) with 530 nm LP filter. The data were present as the mean fluorescence intensity ± SEM.

### Determination of protein levels by Western blotting

The procedure for western blotting analysis of proteins was reported by Zhao *et al*.^52^. Briefly, sperm cells were lysed in RIPA buffer containing the protease inhibitor cocktail from Sangong Biotech, Ltd. (Shanghai, China). Protein concentration was determined by BCA kit (Beyotime Institute of Biotechnology, Shanghai, China). Goat anti-glyceraldehyde 3-phosphate dehydrogenase (GAPDH) (Cat #: sc-48166, Santa Cruz Biotechnology, Inc., Dallas, Texas, USA) was used as a loading control. The other primary antibodies (Abs) were purchased from Beijing Biosynthesis Biotechnology CO., LTD, (Beijing, China) (Table S1). Secondary donkey anti-goat Ab (Cat no.: A0181) was purchased from Beyotime Institute of Biotechnology (Shanghai, P.R. China), and goat anti-rabbit (Cat no.: A24531) Abs were bought from Novex^®^ by life technologies (USA). Fifty micrograms of total protein per sample were loaded onto 10% SDS polyacrylamide electrophoresis gels. The gels were transferred to Polyvinylidene Fluoride (PVDF) membrane at 300 mA for 2.5 hr at 4 °C. Then, the membranes were blocked with 5% BSA for 1 hr at RT, followed by three washes with 0.1% Tween-20 in TBS (TBST). The membranes were incubated with primary Abs diluted with 1:500 in TBST with 1% BSA overnight at 4 °C. After three washes with TBST, the blots were incubated with the HRP-labeled secondary goat anti-rabbit or donkey anti-goat Ab respectively for 1 hr at RT. Then, the blots were imaged after three washes.

### Detection of protein levels and location in spermatozoa using immunofluorescent staining

Hurtado de Llera *et al*.^53^ have reported the methodology for immunofluorescent staining of spermatozoa. After 24 hr treatment boar spermatozoa were fixed in 4% paraformaldehyde for 1 hr, then the cells were spread onto poly-L-lysine coated microscope slides and air-dry. After three washings with PBS (5 min each) spermatozoa were incubated with 2% (vol/vol) Triton X-100 in PBS for 1 hr at RT. Then, after three washes with PBS, the cells were blocked with 1% (wt/vol) BSA and 1% goat serum in PBS for 30 min at RT, then incubation with primary antibodies PI3K, p-AKT and ERK (1:100) diluted in blocking solution overnight at 4 °C. The following morning, after three washes with PBS Tween 20 (0.5%) the slides were incubated with Alexa Fluor 546 goat anti-rabbit IgG (1:200) for 30 min in darkness at RT. The negative controls samples were incubated with secondary antibody and without primary antibody. Slides were washed with PBS Tween-20 three times and then incubated with DAPI (4.6-diamidino-2-phenylindole hydrochloride, 100 ng/ml) as nuclear stain for 5 min. After brief wash with ddH_2_O, the slides were covered with an anti-fading mounting medium (Vector, Burlingame, USA). Fluorescent images were obtained with Leica Laser Scanning Confocal Microscope (LEICA TCS SP5 II, Germany).

### Measurement of Na^+^/K^+^-ATPase activity

The activity of Na^+^/K^+^-ATPase was determined by the kit from Nanjing Jiancheng Biochemistry Co. (Nanjing, China) following the manufacturer’s instruction^54^. Briefly, after 24 hr treatment, the spermatozoa were collected and lysed in 0.9% NaCl. Then the enzyme activity in the lysate was determined spectrophotometrically with the kit. The protein concentration was measured by the BCA method.

### ATP rescue experiment

To test whether ATP can rescue the inhibitory effect of Na_2_S and/or NH_4_Cl on boar spermatozoa motility, 1 or 2 mM ATP (Cat #:10519979001; Sigma-Aldrich Co. LLC) was added to the incubation solution when the spermatozoa were treated with Na_2_S/NH_4_Cl^55^. Then after a 24 h treatment, spermatozoa motility was determined using the CASA method.

### Statistical analysis

The data were statistically analyzed by SPSS statistical software (IBM Co., NY) using ANOVA. Comparisons between groups were tested by One-Way ANOVA analysis and the LSD test. All groups were compared with each other for every parameter (mean ± SEM). Differences were considered significant at *p* < 0.05.

## Additional Information

**How to cite this article**: Zhao, Y. *et al*. Hydrogen Sulfide and/or Ammonia Reduces Spermatozoa Motility through AMPK/AKT Related Pathways. *Sci. Rep.* **6**, 37884; doi: 10.1038/srep37884 (2016).

**Publisher's note:** Springer Nature remains neutral with regard to jurisdictional claims in published maps and institutional affiliations.

## Supplementary Material

Supplementary Information
