# Supplementary material for: Hydrogen Sulfide and/or Ammonia Reduces Spermatozoa Motility through AMPK/AKT Related Pathways
Source: Sci Rep. 2016 Nov 24;6:37884. doi: 10.1038/srep37884 (PMC5121643; doi:10.1038/srep37884)
Supplement: Supplementary Information [file srep37884-s1.doc]

**Hydrogen Sulfide and/or Ammonia Reduces Spermatozoa Motility through AMPK/AKT Related Pathways**

Yong Zhao^1,2#^, Wei-Dong Zhang^2,3#^, Xin-Qi Liu^2^, Peng-Fei Zhang^2,3^, Ya-Nan Hao^2^, Lan Li^3^, Liang Chen^1^, Wei Shen^3^, Xiang-Fang Tang^1^, Ling-Jiang Min^3^, Qingshi Meng^1^, Shu-Kun Wang^1^, Bao Yi^1^, Hong-Fu Zhang^1*^

^1^State Key Laboratory of Animal Nutrition, Institute of Animal Sciences, Chinese Academy of Agricultural Sciences, Beijing 100193, China

^2^College of Chemistry and Pharmaceutical Sciences, Qingdao Agricultural University, Qingdao 266109, P. R. China;

^3^Key Laboratory of Animal Reproduction and Germplasm Enhancement in Universities of Shandong, Qingdao 266109, P. R. China.

*Address correspondence to:

Hong-Fu Zhang, Ph.D., Professor

State Key Laboratory of Animal Nutrition

Institute of Animal Sciences

Chinese Academy of Agricultural Sciences

Yuanmingyuan West Road 2, Haidian Distract

Beijing 100193, China

Tel: +86-10-62819432

Fax: +86-10-62819432

Email: zhanghongfu@caas.cn

**^#^ Co-first author**

**Table S1.** Primary antibody information

| **Gene symbol** | **Name** | **Cat. #** | **Predicted size** | **Source (Animal)** | **Company** |
| --- | --- | --- | --- | --- | --- |
| GAPDH | glyceraldehyde-3-phosphate dehydrogenase | sc-48166 | 37kd | Goat (polyclonal) | Santa Cruz Biotechnology, Inc. |
| Bax | BCL2-Associated X | bs-4564R | 21kd | Rabbit (polyclonal) | Beijing Biosynthesis Biotechnology CO. |
| Caspase 8 | Caspase 8 | bs-0052R | 12/55kd | Rabbit (polyclonal) | Beijing Biosynthesis Biotechnology CO. |
| Caspase 3 | Caspase 3 | bs-0081R | 28kd | Rabbit (polyclonal) | Beijing Biosynthesis Biotechnology CO. |
| Bcl-xl | Bcl-xl | bs-1336R | 26kd | Rabbit (polyclonal) | Beijing Biosynthesis Biotechnology CO. |
| Bcl-2 | Bcl-2 | bs-4563R | 26kd | Rabbit (polyclonal) | Beijing Biosynthesis Biotechnology CO. |
| SOD | super oxide dismutase | bs-1080R | 22kd | Rabbit (polyclonal) | Beijing Biosynthesis Biotechnology CO. |
| GPX | glutathine peroxidase | bs-3882R | 22kd | Rabbit (polyclonal) | Beijing Biosynthesis Biotechnology CO. |
| Catalase | Catalase | bs-2302R | 58kd | Rabbit (polyclonal) | Beijing Biosynthesis Biotechnology CO. |
| AMPK alpha-1 | AMP activated kinase | bs-1115R | 60kd | Rabbit (polyclonal) | Beijing Biosynthesis Biotechnology CO. |
| phospho-AMPK alpha-1 (Thr172) | phophorylated AMPK | bs-4002R | 60kd | Rabbit (polyclonal) | Beijing Biosynthesis Biotechnology CO. |
| TAK1 | Transforming growth factor (TGF_β_) activated kinase-1 | bs-3585R | 67kd | Rabbit (polyclonal) | Beijing Biosynthesis Biotechnology CO. |
| CaMKKa | Ca^2+^/calmodulin-dependent protein kinase alpha | bs-11247R | 56kd | Rabbit (polyclonal) | Beijing Biosynthesis Biotechnology CO. |
| CaMKKb | Ca^2+^/calmodulin-dependent protein kinase beta | bs-6253R | 55kd | Rabbit (polyclonal) | Beijing Biosynthesis Biotechnology CO. |
| LKB1 | LKB1 | bs-3948R | 48kd | Rabbit (polyclonal) | Beijing Biosynthesis Biotechnology CO. |
| ATPase5a | ATP synthetase 5a | bs-2435R | 13kd | Rabbit (polyclonal) | Beijing Biosynthesis Biotechnology CO. |
| ATPase5b | ATP synthetase5b | bs-8600R | 51kd | Rabbit (polyclonal) | Beijing Biosynthesis Biotechnology CO. |
| p-PI3K | phosphorylated Phosphoinositide 3-kinase | bs-5571R | 80kd | Rabbit (polyclonal) | Beijing Biosynthesis Biotechnology CO. |
| AKT1 | protein kinase B | bs-0115R | 56kd | Rabbit (polyclonal) | Beijing Biosynthesis Biotechnology CO. |
| p-AKT | phosphorylated AKT | bs-2720R | 56kd | Rabbit (polyclonal) | Beijing Biosynthesis Biotechnology CO. |
| p-ERK: | phosphorylated Extracellular signal-regulated kinases | bs-3292R | 41kd | Rabbit (polyclonal) | Beijing Biosynthesis Biotechnology CO. |
| ERk1+ERK2 | Extracellular signal-regulated kinases | bs-0022R-HRP | 42kd | Rabbit (polyclonal) | Beijing Biosynthesis Biotechnology CO. |
| PTEN | Phosphatase and tensin homolog deleted on chromosome 10 (PTEN) | bs-0686R | 44kd | Rabbit (polyclonal) | Beijing Biosynthesis Biotechnology CO. |
| p-PTEN | phosphorylated PTEN | bs-3351R | 44kd | Rabbit (polyclonal) | Beijing Biosynthesis Biotechnology CO. |
